# Oligomerization and ATP stimulate condensin-mediated DNA compaction

**DOI:** 10.1038/s41598-017-14701-5

**Published:** 2017-10-27

**Authors:** Ross A. Keenholtz, Thillaivillalan Dhanaraman, Roger Palou, Jia Yu, Damien D’Amours, John F. Marko

**Affiliations:** 1 Department of Molecular Biosciences, Northwestern University, Evanston, IL 60208 USA; 2 Institute for Research in Immunology and Cancer, Université de Montréal, Montréal, Québec, H3C 3J7 Canada; 3 College of Life Sciences, Qingdao University, Qingdao, 266071 China; 4 Department of Cellular and Molecular Medicine, University of Ottawa, Ottawa, Ontario, K1H 8M5 Canada; 5 Department of Physics and Astronomy, Northwestern University, Evanston, IL 60208 USA

## Abstract

Large-scale chromatin remodeling during mitosis is catalyzed by a heteropentameric enzyme known as condensin. The DNA-organizing mechanism of condensin depends on the energy of ATP hydrolysis but how this activity specifically promotes proper compaction and segregation of chromosomes during mitosis remains poorly understood. Purification of budding yeast condensin reveals that it occurs not only in the classical heteropentameric “monomer” form, but that it also adopts much larger configurations consistent with oligomerization. We use a single-DNA magnetic tweezers assay to study compaction of DNA by yeast condensin, with the result that only the multimer shows ATP-enhanced DNA-compaction. The compaction reaction involves step-like events of 200 nm (600 bp) size and is strongly suppressed by forces above 1 pN, consistent with a loop-capture mechanism for initial binding and compaction. The compaction reactions are largely insensitive to DNA torsional stress. Our results suggest a physiological role for oligomerized condensin in driving gradual chromatin compaction by step-like and slow “creeping” dynamics consistent with a loop-extrusion mechanism.

## Introduction

**Figure 1:**
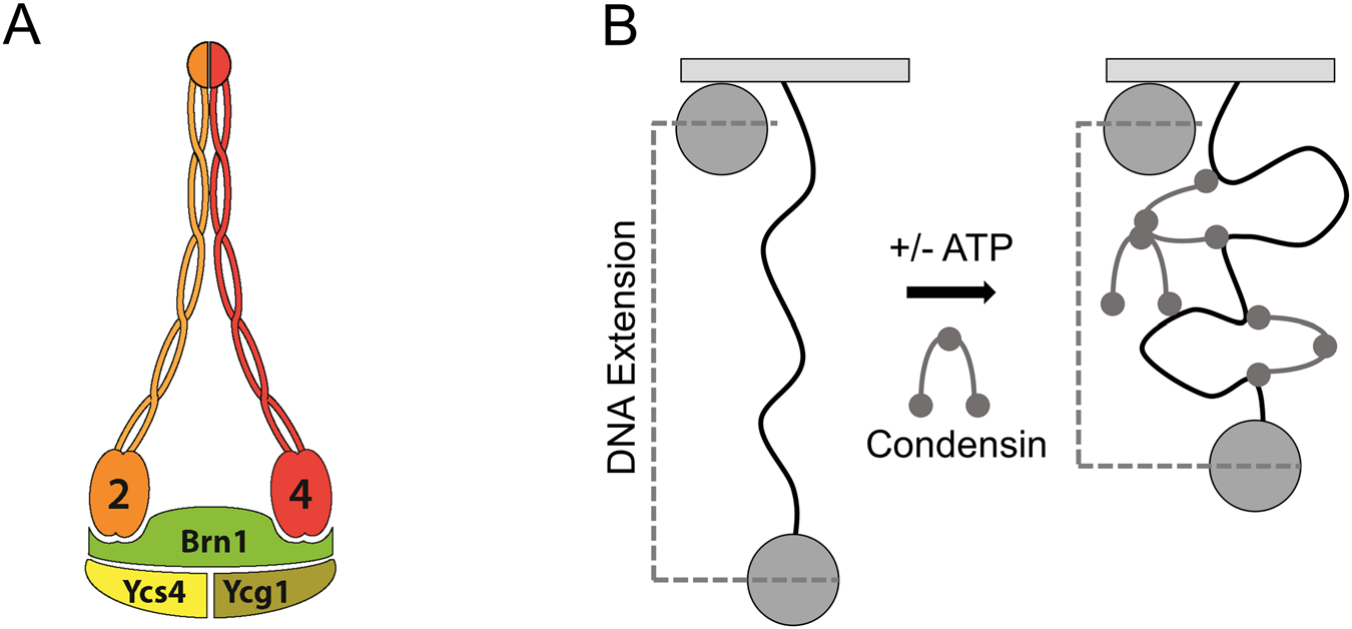
Yeast condensin complex and magnetic-tweezers single-DNA compaction assay. (**A**) Yeast condensin complex. Sketch of yeast condensin pentamer containing Smc2 and Smc4 subunits, complexed with the kleisin Brn1, as well as Ycg1 and Ycs4. (**B**) Magnetic tweezers single-DNA compaction assay. A single linear 10 kb molecule of double-stranded DNA is tethered at one end to the surface of a glass coverslip via digoxigenin and anti-digoxigenin interactions and at the other end to a streptavidin coated paramagnetic bead via a biotin linkage. The paramagnetic bead is held under constant 0.45 pN force by a magnet underneath the flow cell. A surface bead attached to the glass coverslip acts as a reference point for the bead attached to the DNA tether. As condensin is added to the flow cell, the protein molecules bind to and compact DNA.

Chromosome compaction is a dynamic process responsible for the formation of visible chromosomes in mitosis and meiosis. During the cell cycle, chromatin undergoes several morphological changes from an amorphous mass of DNA in interphase to a fully compacted structure at metaphase. Condensin, a large protein complex containing two Structural Maintenance of Chromosomes (SMC) proteins (Fig. 1A), plays a central role in shaping chromatin architecture and dynamics during cell division^1,2^. More recently, several lines of evidence have suggested that the activity of condensin is not only limited to chromosome compaction, but is also required for a broad range of cellular functions including cell differentiation^3^, development and gene expression^1,4^. Despite these important roles for condensin, its mechanism of action is poorly understood. In particular, how condensin binds to DNA and stimulates chromatin compaction remains an important question in cell biology.

There are three conserved families of SMC complexes in eukaryotes: cohesin, condensin, and the Smc5–6 complex^5–8^. These complexes are built around a heterodimeric core of SMC proteins. For condensin, these are the Smc2-Smc4 subunits, whereas cohesin contains Smc1-Smc3, and the last member of this family is named after its core components, Smc5 and Smc6. SMC proteins have N- and C-terminal globular domains that contain Walker A and B motifs, respectively. These motifs form together a bipartite ATP-binding site or ATPase head domain. The amino- and carboxy-terminal domains of SMC proteins are connected in the folded molecule to a central hinge domain by a long coiled-coil region^9^. The hinge regions of non-identical pairs of SMC molecules interact together to form a V-shaped SMC heterodimer. The ATPase heads of SMC proteins are then connected through separate interactions with a non-SMC subunit of the “Kleisin” family (Brn1/CapH for condensin and Mcd1/Scc1/Rad21 for cohesin). The end result is the formation of ring-like structures that are believed to play a central role in constraining chromatin configuration during cell division.

To perform their molecular functions, it is possible that SMC complexes form higher order structures or oligomers. Extant observations supporting oligomerization of SMC complexes arise largely from *in vitro* studies on prokaryotic SMC proteins. When the purified *E*. *coli* MukB homodimer and MukBEF complex were subjected to electron microscopic studies, images revealed fibres and rosette-like configurations consistent with oligomer formation. Further, the authors suggested that the oligomerization of MukBEF is mediated by the intermolecular interactions between MukE and MukF because the MukB homodimer alone is rarely seen as multimeric^10^.

Protein volume, shape and interaction studies by atomic force microscopy also showed oligomeric structures with the *B*. *subtilis* SMC complex in the presence of its ScpA and ScpB subunits. Interestingly, this study also confirmed that ScpA binds directly to SMC protein, whereas ScpB binds SMC in presence of ScpA, suggesting that ScpA-B plays a key role in organisation of SMC oligomers^11^. Likewise, analysis of yeast condensin Smc2-Smc4 subunits revealed that the 296 kDa dimer elutes during size exclusion chromatography at positions corresponding in mass to ~0.2–1.2 MDa, indicating possible oligomerization of condensin’s heterodimeric SMC core^12^. Similar observations were made with purified yeast condensin holoenzyme^13^.

Recent genetic analyses of interallelic complementation patterns in yeast has led to the proposal of oligomeric forms of cohesin in cells. In this study, the authors successfully showed that cells harbouring individual *mcd1* or *smc3* mutant alleles are not viable, but co-expression of these otherwise defective alleles is associated with a recovery of cell viability and functional sister-chromatid cohesion^14^. In addition, experiments with *Xenopus* nuclear extracts have suggested that there are interactions between condensin monomers^15^. Collectively, these results are consistent with a mechanism of SMC proteins oligomerization whereby two or more SMC complexes directly interact to perform their functions.

Single-molecule DNA compaction experiments have been used to examine the function of SMC complexes, with results suggesting a role for oligomerization or cooperativity. Experiments using condensin isolated from *X*. *laevis* egg extracts suggested that compaction of DNA by this enzyme is the net effect of the co-operative action between multiple condensin molecules^16^. Schematically similar studies of *E*. *coli* MukBEF complexes led to similar conclusions that DNA compaction involved SMC oligomerization^17^. Quite recent studies of bsSMC also suggest a role for ATP-dependent inter-complex cooperativity in single-molecule DNA-compaction experiments^18^.

In this study, we investigate the impact of budding yeast condensin’s oligomerization state on its DNA compaction activity. Here we report experiments with condensin purified from asynchronous yeast cultures, an enzyme preparation containing both mitotic and interphase post-translational modifications. We show that condensin purified from asynchronous budding yeast cultures^13^ exists as two distinct populations corresponding in apparent mass to monomers and multimers of the enzyme complex. Then, we use a single-molecule magnetic tweezers-based DNA-compaction assay (Fig. 1B) to show that the multimeric form of condensin is more active than the monomer in DNA compaction reactions in the presence of physiological levels of ATP.

## Materials and Methods

### Protein purification, stability and oligomerization experiments

The condensin holoenzyme was purified according to the method described in St-Pierre *et al*.^13^. To assess the stability of the monomeric condensin, protein fractions corresponding to the monomeric peak in Superose 6 chromatogram were pooled and concentrated using 100 kDa Amicon Ultra-15 centrifugal filters (Millipore) at 4 °C. The concentrated sample was again resolved on the Superose 6 10/300 size exclusion column (GE Healthcare). The enzyme purified using this approach is not a fully-phosphorylated form of condensin as it was isolated from an asynchronous culture of yeast. *In vitro* phosphorylation experiments for condensin were performed as previously published^13,19^. Note that the multimeric fraction of condensin contains a small proportion of monomers since there is a degree of overlap between their elution peaks in SEC.

To study the oligomerization of yeast condensin, protein fractions obtained after Streptactin chromatography were divided into 2 equal parts. One part was diluted 30-fold with Superose 6 buffer (10 mM Tris-HCl [pH 8], 50 mM NaH_2_PO_4_ [pH 7.8], 500 mM NaCl, 0.05% Tween 20, 1 mM EDTA, 2 mM β-mercaptoethanol) whereas the other part of the protein was left undiluted. Both samples were incubated at 4 °C for 36 hours allowing sufficient time for monomer-to-multimer conversion. After 36 hours, both samples were concentrated to 1 ml and resolved on Superose 6 10/300 size exclusion column. Protein fractions corresponding to monomer and multimer peaks were analyzed on 4–12% Criterion™ XT Bis-Tris gels (BioRad). To evaluate condensin abundance, Coomassie-strained gels were scanned at high resolution and bands corresponding to condensin subunits were subjected to pixel analysis using Image J software. The ATPase-defective form of condensin contained Smc4-K191M.

### Condensin ATP-dependent oligomerization assay

To study the effect of ATP on oligomerization of yeast condensin, monomeric condensin fractions were pooled, concentrated, and divided into two parts. One part was supplemented with 1 mM ATP, whereas the other part was left untreated. The samples were incubated overnight at 4 °C to allow sufficient time for changes in condensin oligomerization state. After this incubation, both the samples were resolved by size exclusion chromatography, as previously described.

### ATP-binding assay

Binding of ATP to yeast condensin was performed using the UV crosslinking procedure described in^20^, with  slight modifications. In brief, mixtures of 350 nM multimeric and 325 nM monomeric yeast condensin were prepared in 12.5 µl reaction buffer containing 10 mM HEPES (pH 8.0), 1 mM MgCl_2_, 1 mM β-mercaptoethanol, 45 nM [γ -^32^P]ATP (3000 Ci/mmol). The mixtures were transferred to a parafilm wrap on ice, and were exposed to UV irradiation (254 nm) for 10 minutes to induce crosslinking. After crosslinking, protein samples were separated by SDS–PAGE and the radioactive signal was detected using a Typhoon FLA-9500 Bio-Image Analyzer. The ATP-bound signal detected in condensin subunits was normalized to the protein signal detected in the coomassie-stained gels to account for any difference in loading between multimer and monomer samples. A *t*-test (calculated using Prism 6.0 [GraphPad]) was performed to determine if the normalized ATP-bound signals of monomeric and multimeric condensins were statistically different.

### DNA constructs for single-DNA experiments

Linear DNA fragments used in single-molecule supercoiling relaxation experiments were derived from the plasmid pNG1175 (9702 bp), a slightly modified version of pFOS-1 (9691 bp, New England Biolabs)^21^. pNG1175 was linearized by cutting at nearby Spe and ApaI restriction sites; the resulting linear molecule was ligated to ≈900 bp PCR products carrying either biotinylated or digoxigenin-labeled nucleotides, prepared with SpeI and ApaI-compatible ends, respectively. The resulting linear constructs were 11.4 kb in length, with roughly 900-bp of biotin- and digoxigenin-labeled DNA at their ends, allowing multiple tethering of the ends to streptavidin- or anti-digoxigenin-coated surfaces. The multiple tethers constrain the two DNA strands sufficiently that they may be supercoiled by rotation of the magnetic particle.

### Single-DNA experiments

Flow cells were assembled for each experiment and contained 2.8 μm streptavidin coated paramagnetic beads (Invitrogen Dynabeads, M280) tethered to the surface of an anti-digoxigenin coated glass coverslip via a linear pNG1175 DNA molecule with biotinylated and digoxigenin-labeled ends (Fig. 1B).

Flow cell contents were viewed with a bright field microscope and a 100× 1.3 NA immersion oil objective (Olympus). Translation in the z direction of a permanent magnet under the objective stage controlled the force on the bead while 360° rotations of the magnet controlled the linking number of the tethered DNA molecule. Bead position in three dimensions was tracked with custom lab-written software, which uses an untethered bead nonspecifically bound to the glass surface as a reference point. Position fluctuations in the x-y plane were used to calibrate the force on the tethered beads while changes in the z direction relative to the reference bead were used to measure the tether extension^22^. Experiments recorded DNA extension for approximately 1000 sec, at approximately 100 measurements per second.

Single-molecule experiments were carried out in 1X assay buffer containing 50 mM potassium glutamate (KGlu), 10 mM HEPES, pH 7.5 and 1 mM MgCl_2_ at 30 °C. An additional 1 mM ATP was supplemented where noted. Condensin was added to a 150 μL mixture of 1X assay buffer for a final concentration of 5 nM and immediately added to flow cells with tethers initially held at 4 pN force, with force reduced to 0.45 pN (or other forces as indicated for force-titration experiments) following addition of enzyme solution to the flow cell (a process described below as “flow-through”).

There are variations in tether length due to adhering of varied amounts of the 900 bp (300 nm) labeled ends, as well as due to random variation in the bead sizes, position on the bead of DNA tethering, and bead optical properties, which affect the precise location of the focal plane that determines our inferred position of the beads in the vertical direction. These effects lead to variation of initial length in the few hundred nanometer range as observed.

### Data Availability

The datasets generated during and/or analyzed during the current study are available from the corresponding author on request. Note that data presented in the quantitation figures of the main manuscript are included in the Supplementary Information (Supplementary Worksheet).

## Results

### Purified yeast condensin exists in two stable oligomerization states

**Figure 2:**
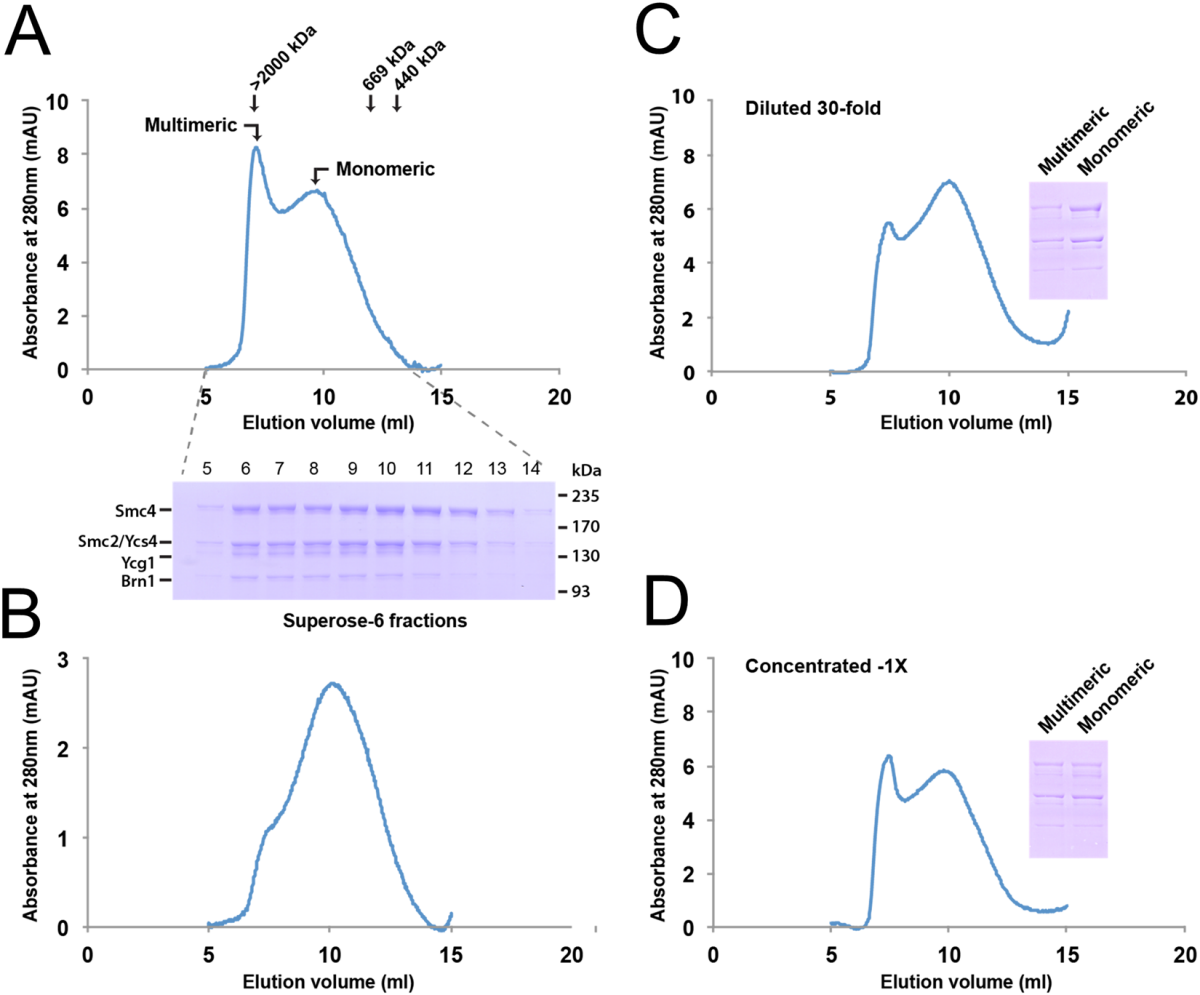
Oligomerization state of condensin after purification. (**A**) Purification of condensin holoenzyme from yeast. Condensin was purified from whole cell lysate using a multi-step purification procedure. Specifically, purification was carried out using sequential steps of nickel-NTA and StrepTactin affinity chromatography, followed by gel filtration on Superose 6 column. The elution profile of condensin from the last step of purification is shown in the top graph, whereas the purity of condensin subunits is shown in the Coomassie-stained gel below the graph. The elution profile shows 2 major peaks of condensin corresponding to multimeric (fractions 6–7 mL) and monomeric (fractions 9–13 mL) sizes for the enzyme (in accord with data in^13^). (**B**) Stability of monomeric condensin. Following size exclusion chromatography, fractions containing monomeric condensin were pooled, concentrated and resolved again on Superose 6 column. Condensin elutes mostly as a monomeric species after the second round of SEC and little conversion to a multimeric state is observed. (**C–D**) Oligomerization of yeast condensin is concentration dependent. Purified yeast condensin was diluted 30-fold (panel C) or left undiluted (panel D) and kept at 4 °C for 36 hours to allow multimer–monomer conversion. After 36 hours, the sample was run on a Superose 6 column. The elution profile of the diluted fractions (panel C) shows a partial conversion of multimeric condensin to a monomeric state. In contrast, the multimeric form of condensin (panel D) remained largely unaffected in the undiluted condensin preparation. Peak fractions containing monomeric and multimeric condensins were resolved by SDS-PAGE after SEC and condensin subunits were revealed by Coomassie staining (insets).

Previous work has indicated that condensin elutes as two major populations during size exclusion chromatography (SEC)^13^. The early elution peak corresponds in mass to multimeric complexes of approximately 2 MDa, whereas the late elution peak has an apparent molecular mass similar to that of a monomeric complex (≈600 kDa; the calculated molecular mass of the recombinant complex is 651 kDa). We confirmed this observation and show that all the subunits of yeast condensin are present in both peaks (Fig. 2A; bottom panel). To investigate the stability of monomeric condensin, we pooled the terminal end fractions of the monomeric peak (to minimize contamination with the multimeric enzyme) and kept them at 4 °C for 2 hours, allowing sufficient time for monomer-multimer conversion. The sample was re-run on the same SEC column that was initially used to purify the enzyme. Interestingly, we observed that the monomeric enzyme still eluted mainly as a monomer during the second cycle of SEC (Fig. 2B). The elution profile of the monomeric fraction contained a small shoulder/bump that likely corresponds to a low-level contamination of multimeric complexes in the original monomer fraction (*i.e*., there is a degree of overlap in the elution profiles of both fractions). Together, these results indicate that monomeric condensin is highly stable and there is no quantitative conversion of monomer to multimer state under our experimental conditions.

In order to investigate whether ATP has any effect on protein stability and oligomerization state of condensin, we incubated the monomeric fraction with 1 mM ATP at 4 °C for 16 hours. The sample was subsequently run on the gel filtration column and compared with the mock-treated control. The comparison showed no significant effect of ATP on the conversion of monomeric condensin to its oligomeric state (Fig. S1).

### Oligomerization of yeast condensin is concentration-dependent

We next wanted to investigate whether the multimeric state of condensin was stable over time. However, because of the overlap in the SEC elution profiles of monomeric and multimeric condensins, it was not possible to isolate an absolutely pure multimeric fraction of this enzyme. To address this limitation, we used an approach in which we divided into two halves the Streptactin-purified fraction of the enzyme (which contains a mixed pool of ~30% multimeric and ~70% monomeric condensin). One of the halves was diluted 30-fold with SEC buffer, whereas the other was left undiluted. Both samples were kept at 4 °C for 36 hours, allowing sufficient time for multimer-to-monomer conversion. After 36 hours, both samples were concentrated and analyzed by SEC.

Interestingly, we observed a notable difference between the two elution profiles obtained during the SEC analysis. Compared to the undiluted sample, the relative amount of multimeric condensin was reduced considerably when the enzyme was diluted, and this was associated with a corresponding increase in the monomeric fraction. This result indicates that at lower concentration, the multimeric form of condensin tends to disassociate and form monomers, whereas the oligomerization state of condensin is maintained at higher enzyme concentrations (Fig. 2C,D). To further substantiate this, we monitored the protein content from both peak fractions by SDS-PAGE. Coomassie staining showed a higher concentration of monomeric yeast condensin in the diluted sample as compared to the monomer contained in the undiluted samples. Taken together, these observations indicate that multimeric condensin can convert to its monomeric state, and this conversion is a concentration dependent process that does not require ATP.

### ATP stimulates DNA compaction by multimeric condensin

**Figure 3:**
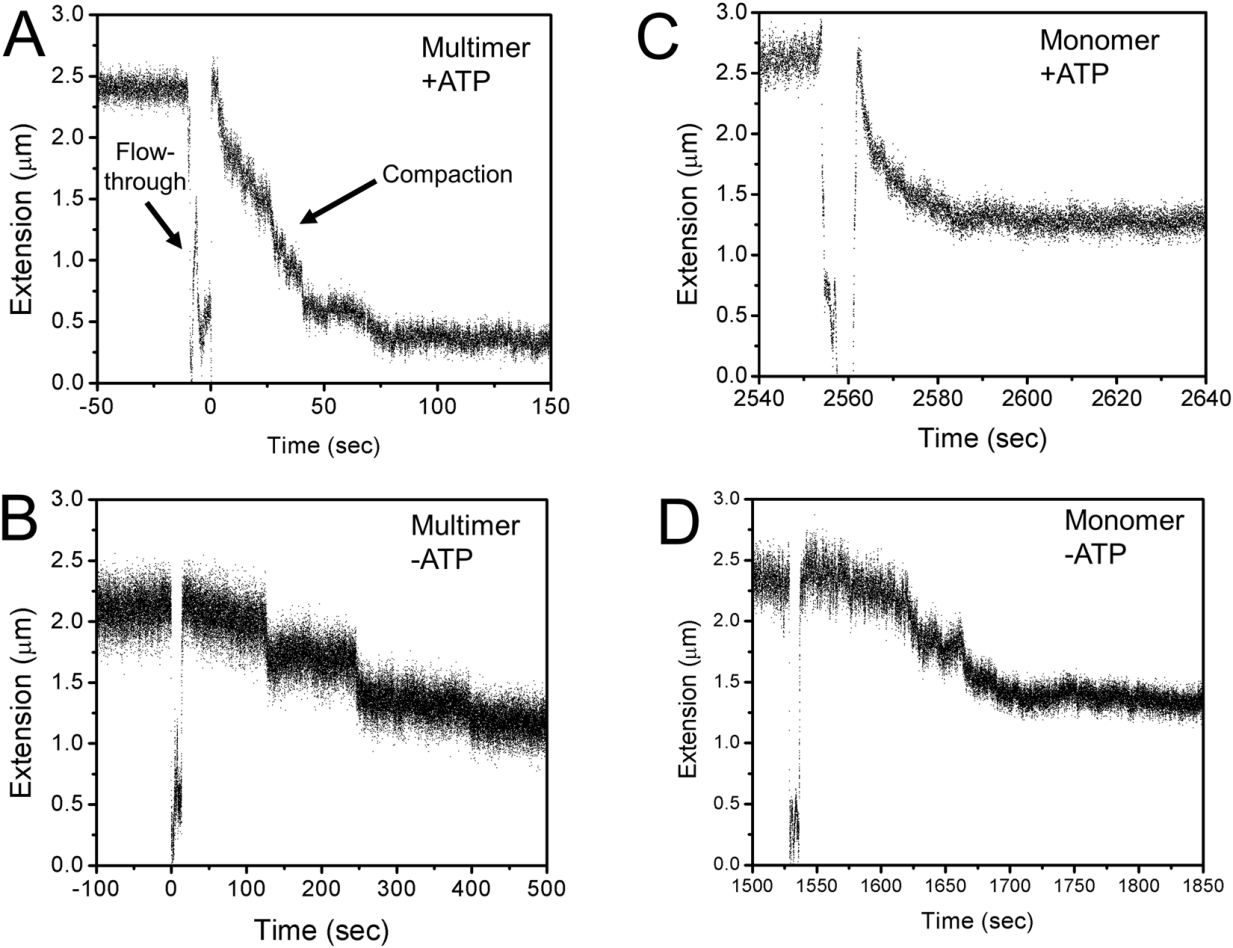
Single molecule magnetic tweezers compaction traces for multimer and monomer condensin with and without ATP. (**A**) Compaction by multimeric condensin with ATP. The readout of each experiment is the length of the DNA tether. Under 0.45 pN force, the naked DNA is extended to ≈2.4 μm. When multimeric condensin is added in the presence of ATP (“flow-through”), the DNA tether is compacted by ≈70%. (**B**) Compaction by multimeric condensin without ATP. Similar to Fig. 3A but without ATP, the DNA tether is compacted by condensin but to a final compaction percentage of ≈45%. (**C**) Compaction by monomeric condensin with ATP. Similar to Fig. 3A but with monomeric condensin instead of multimeric, the DNA tether is compacted by condensin to a final compaction percentage of ≈45%. (**D**) Compaction by monomeric condensin without ATP. Similar to Fig. 3B but with monomeric condensin instead of multimeric, the DNA tether is compacted by condensin to a final compaction percentage of ≈45%.

**Figure 4:**
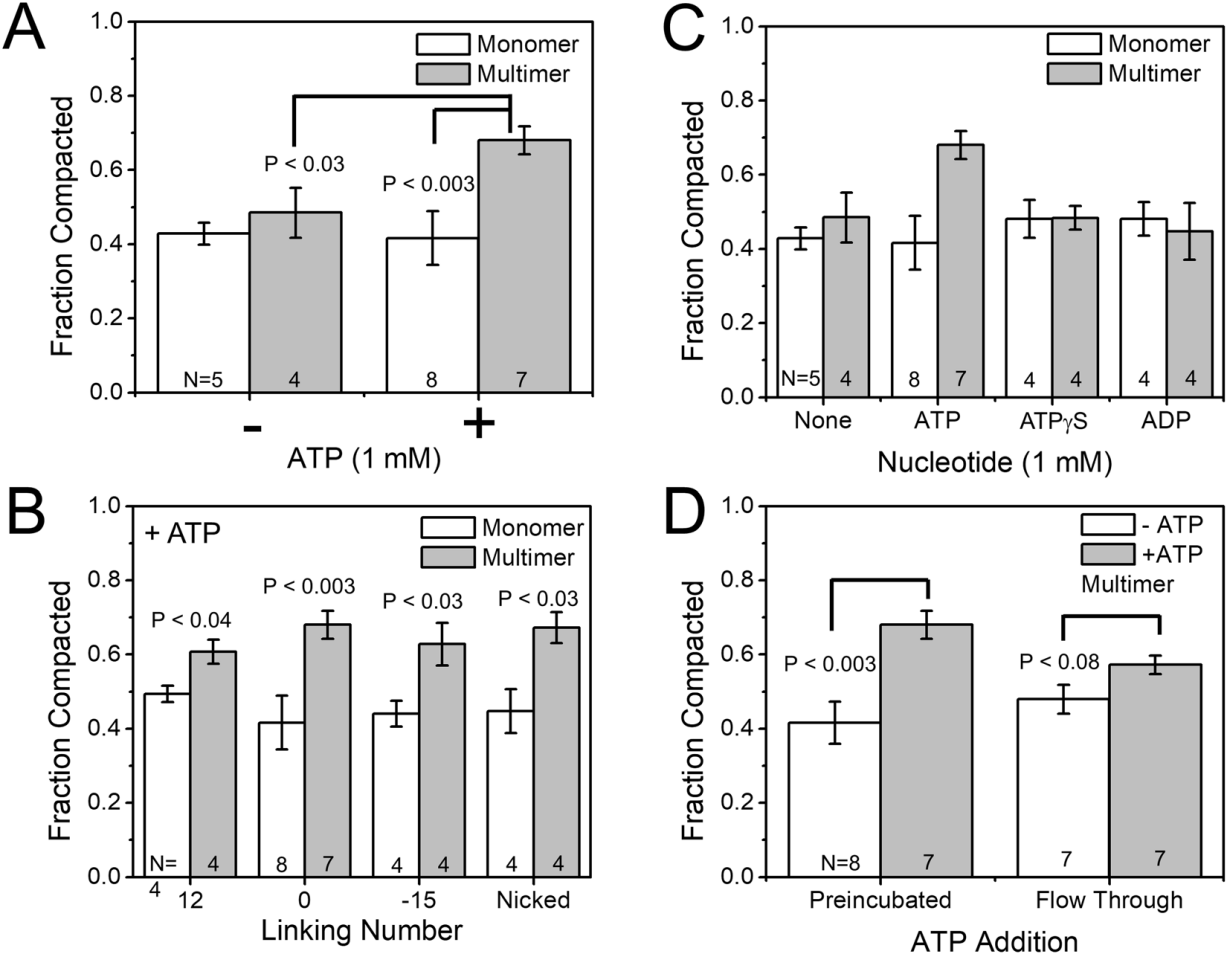
Nucleotide and DNA linking number dependence of compaction. (**A**) ATP and oligomeric state dependence on compaction. Summary of experiments testing the monomeric and multimeric forms of condensin in the presence and absence of ATP (1 mM). Each bar represents at least 4 independent trials (number of trials for each case are indicated by the number N on the bars). Significantly stronger compaction is obtained in the multimer + ATP case relative to the other cases (monomer + ATP, monomer-ATP, multimer-ATP), suggesting that only the condensin multimer is able to use ATP to drive enhanced DNA compaction. 1 mM magnesium chloride was present in each experiment. Data sets for each bar graph are given numerically and shown as scatter plots in Supplementary Worksheet 1. (**B**) Linking number and oligomeric state dependence on compaction. Summary of experiments testing the monomeric and multimeric forms of condensin in the presence of ATP plus magnesium (1 mM each) on DNA tethers of fixed values of linking number, as well for nicked DNA (zero DNA torsional stress). Each bar shows data from at least 4 independent trials. Essentially the same compaction is achieved for each monomer + ATP and multimer + ATP reactions for all cases of DNA torsional stress, indicating that torsional stress plays little role in the compaction reaction. Note that the Lk = 0 data are those shown in Fig. 4A. (**C**) Nucleotide effect on DNA compaction. Either monomeric or monomeric condensin was preincubated with 1 mM ATP, ATPγS, ADP or no nucleotide and used for a DNA compaction assay. each bar represents at least 4 independent trials. The enhanced compaction seen for the multimer + ATP case is absent for the cases of multimer combined with ATP or nonhydrolyzable ATPγS and for all monomer cases, indicating that the enhanced DNA compaction involves ATP hydrolysis. Note that the no-ATP and ATP data are those shown in Fig. 4A. (**D**) ATP incubation and DNA compaction. Multimeric condensin was used for a compaction assay where the condensin was either preincubated with 1 mM ATP, or where buffer plus ATP was flowed in exogenously (“flow-through”) after initial tether compaction with multimeric condensin in the absence of ATP. The significant enhancement of DNA compaction in the preincubated case is reduced in the flow-through case, indicating that presence of ATP at the beginning of the reaction is important to achieving strong DNA compaction. Note that the preincubated case data is the same as that for the multimer shown in Fig. 4A.

Purified yeast condensin eluting at the multimeric position during SEC was added to flow cells containing single-DNA tethers while extension was tracked in real time (Fig. 3A-B; Fig. 3A has the “flow-through”, the addition of condensin and cofactors, marked). Initial experiments at 0.45 pN yielded an extended tether length of ~2.5 μm. This force is similar to *in vivo* conditions of a supercoiled plasmid^23^. When 5 nM multimeric condensin was first incubated with 1 mM ATP and added to the flow cell, tethers were robustly compacted (Fig. 3A), thus establishing an experimental method to monitor condensin mechanism. Typical traces contained a mixture or step and non-step-like compaction events and tethers were routinely compacted to approximately 30% of their initial length (Fig. 4A, rightmost bar; bar graphs show DNA “fraction compacted” which is the complement of final length as a fraction of the initial length, *i.e*., [Fraction Compacted] = 1 − [Final length as a fraction of initial DNA length]; for the multimer + ATP reaction, the fraction compacted was thus 70%. Data sets for each bar graph are shown as scatter plots in the Supplementary Worksheet.).

In contrast, when ATP was not included in the reaction, multimeric condensin (Fig. 3B) showed less robust compaction, with a fraction compacted of 40–50% (N = 4, Fig. 4A, 2nd bar from left). Experiments with 5 nM monomeric condensin showed no difference in the presence (Fig. 4A, leftmost bar, N = 8) or absence (Fig. 4A, 3rd bar from left, N = 5) of ATP with both conditions again leading to a fraction compacted of 40–50%. Thus, ATP stimulated DNA compaction by multimeric condensin significantly relative to -ATP conditions of multimeric condensin (Fig. 4A, p < 0.03) and + ATP conditions of monomeric condensin (Fig. 4A, *p* < 0.003). These differences are not explained by a differential ability of condensin to bind ATP in monomeric or multimeric configurations, as shown by UV crosslinking experiments with radiolabeled ATP (Fig. S2).

Given the differences between the multimer + ATP versus multimer-ATP and monomer ± ATP cases, we examined the kinetics of the reactions. Each time course had a well-defined initial and final DNA extension (Fig. 3) and we measured the time interval between when the compaction was 10% and 90% complete. When we averaged the results over the courses, we obtained compaction completion times which were, within our statistical error, the same for the four reaction types (Fig. S3). We also did not observe a statistically significant dependence of the delay (“dwell time”) between addition of protein and nucleotide and the onset of compaction on multimer/monomer or + ATP/-ATP conditions. Finally, we note that although the 0.45 pN reactions involve primarily compaction steps, we do observe a smaller number of “opening” or decompaction steps, as can be observed in Fig. 3.

### Yeast condensin shows no dependence of compaction on DNA torsional stress

In order to probe deeper into the substrate preference for condensin, a series of linking number experiments were performed, using a method similar to that previously used for studying the DNA torsional stress (torque) dependence of DNA compaction by yeast cohesin^24^. Supercoilable tethers were set to a linking number of either 0 (the data set of Fig. 4A), −12, or + 15; in an additional series of trials nicked DNAs were used. These nonzero linking number values are just slightly below the buckling point where plectonemic DNA begins to form, and thus establish appreciable DNA torque without reducing DNA length via plectoneme formation^24^. All experiments were performed in the presence of ATP, with either monomeric or multimeric condensin. In all cases (Fig. 4B), the multimeric condensin drove a fraction compacted of 60–70% while monomeric condensin achieved a fraction compacted of only 40–50%. This indicated that the monomeric and multimeric compaction reactions were essentially insensitive to DNA torque.

### ATP is required for enhanced DNA compaction

After determining that ATP stimulates multimeric condensin and that condensin has no linking number preference for its substrates, we performed a number of control experiments to delineate the nucleotide effect on DNA compaction. Experiments with ATP included preincubation with 1 mM nucleotide and 1 mM MgCl_2_. Thus, the experiments were repeated where ATP was replaced with either ATPγS or ADP. In each case (Fig. 4C; data for no nucleotide and ATP are the same as that in Fig. 4A), both monomeric and multimeric condensin generated a fraction compacted of 40–50%, suggesting that ATP hydrolysis, not binding, is required for enhanced DNA compaction.

We also carried out a set of experiments with a condensin complex containing a version of Smc4 with a mutation inactivating a key residue of its Walker A motif (Lys191) required for ATP hydrolysis in all SMC proteins (e.g., ref.^25^). This mutant complex, like wild-type condensin, could be separated into monomeric and multimeric fractions. We carried out single-DNA compaction experiments on the multimeric fraction with and without ATP (Fig. S4) at an applied force of 0.45 pN, with the result that there was compaction nearly identical to that of the wild-type multimeric complex in the absence of ATP (Fig. 4A, multimer –ATP bar), and to that of nonhydrolysable ATP (Fig. 4C, multimer + ATPγS). This result indicates that ATP hydrolysis is likely responsible for the enhanced compaction observed for the multimer + ATP reactions.

Next, we sought to identify how ATP hydrolysis stimulates enhanced DNA compaction by multimeric condensin. In contrast to initial experiments where ATP was preincubated with condensin, DNA tethers were first compacted to equilibrium with multimeric condensin in the absence of ATP, which led to a fraction compacted of 48 ± 4%. Next, buffer containing 1 mM ATP was added to the flow cell and the DNA-resident condensin was allowed to continue compaction; an increase in compaction was observed, with a final fraction compacted of 57 ±2% (*p* = 0.08, Fig. 4D, rightmost two bars). This effect is weaker than that when ATP was included in the initial reaction (41 ± 6% compaction in the absence of ATP and 68 ± 4% for condensin preincubated with 1 mM ATP, *p < *0.003, Fig. 4D leftmost two bars show multimer data of Fig. 4A for comparison). This result indicates that exogenous ATP added to the result of the –ATP condensin-DNA reaction can stimulate continued compaction, but not to the same degree as preincubation.

### DNA compaction by condensin is suppressed by applied forces larger than 1 pN

In general, DNA-compaction reactions can be expected to be suppressed by force, since this increases the work done during compaction. It should be noted that a DNA tether starts to strongly fluctuate in shape for forces below 0.5 pN, and becomes quite strongly tensed for forces above 0.5 pN^26^; notably, DNA looping is strongly suppressed for forces above 1 pN. DNA tension in the 0.5 pN range is also that found in physiologically supercoiled DNA molecules, and is a rather typical level of DNA tension thought to be found *in vivo* ^23^.

**Figure 5:**
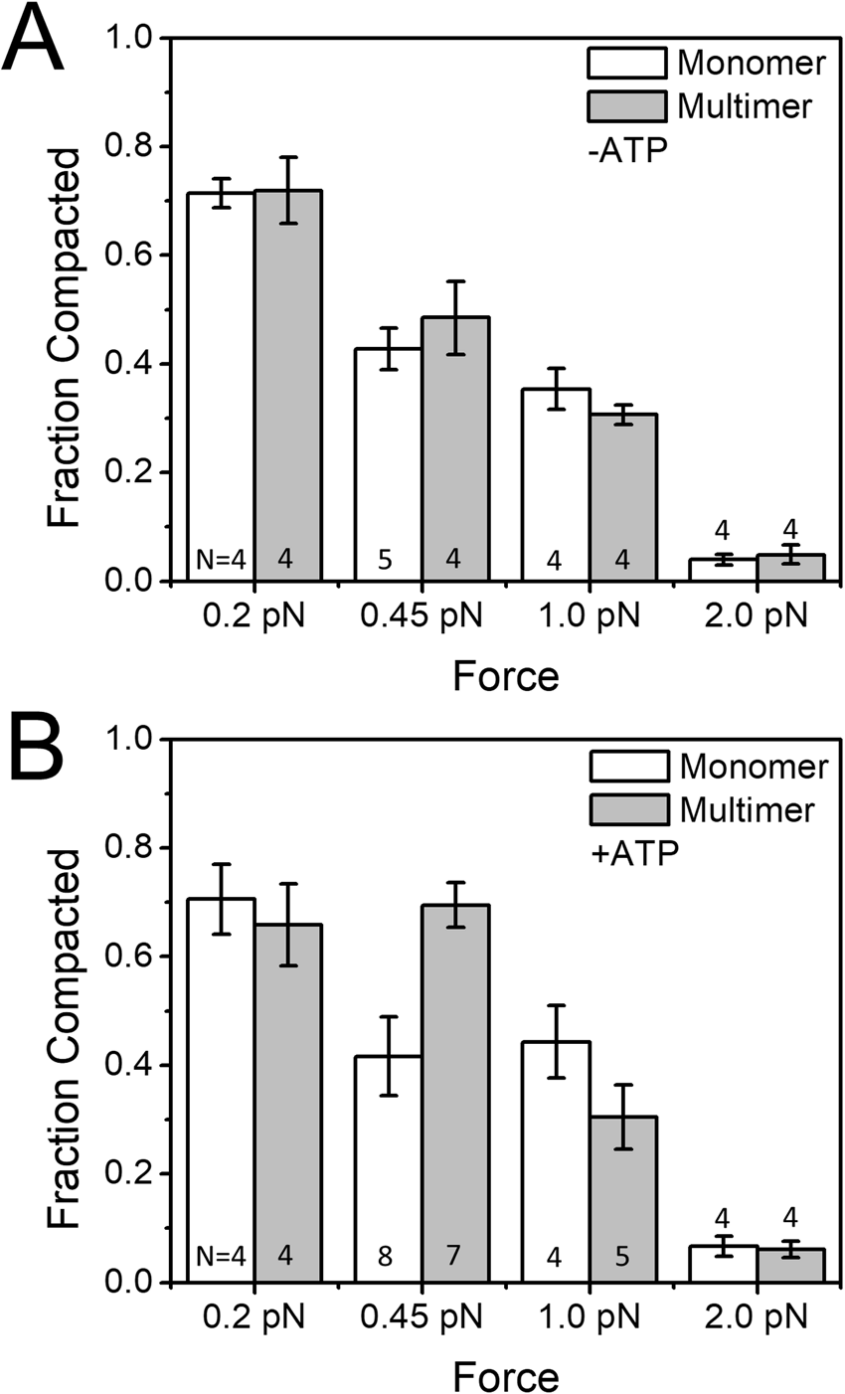
Nucleotide and force dependence of compaction. (**A**) Force dependence on DNA compaction without ATP. DNA compaction was measured at forces of 2.0 pN, 1.0 pN, and 0.45 pN for both monomeric and multimeric condensin, for monomer and multimer in the absence of ATP. Increasing forces suppress the compaction reaction, and there is little difference between monomer and multimer cases. For low forces (0.2 pN) there is strong compaction; for high forces (2 pN) there is little compaction. Both monomer and multimer are capable of generating ≈1 pN compaction forces, consistent with a random-loop-capture mechanism. Note that the 0.45 pN data are those shown in Fig. 4A. (**B**) Force dependence on DNA compaction with ATP. DNA compaction was measured at forces of 2.0 pN, 1.0 pN, and 0.45 pN for both monomeric and multimeric condensin, for monomer and multimer in the presence of ATP (1 mM, with 1 mM Mg^2+^). For low forces (0.2 pN), the reaction is similar to the –ATP reaction, with both monomer and multimer achieving strong compaction; large forces (2 pN) abrogate compaction as in the –ATP case. However, for the intermediate force of 0.45 pN, there is appreciable enhancement of DNA compaction by the multimer + ATP over that of the multimer without ATP, and over the monomer either with or without ATP. Enhanced compaction by the multimer therefore requires hydrolyzable ATP and DNA tension ≈0.5 pN. Note that the 0.45 pN data are those shown in Fig. 4A.

We carried out a series of experiments with and without 1 mM ATP with varied forces applied to the DNA tether (Fig. 5A, B show results for the –ATP and + ATP cases respectively; data for 0.45 pN is that of Fig. 4A). For forces of 0.2 pN we observed a very strong DNA compaction reaction, with little difference between monomeric and multimeric condensin, for reactions with or without ATP. Forces of 1 pN appreciably suppressed DNA compaction by multimeric condensin, but had less effect on the less complete monomeric condensin reaction. DNA tension of 2 pN strongly suppressed DNA compaction by both monomeric and multimeric condensin. Thus both monomeric and multimeric reactions with and without ATP displayed suppression by applied force, although with slightly different force dependences.

### Step size measurements show a mixture of discrete and non-discrete compaction events

**Figure 6:**
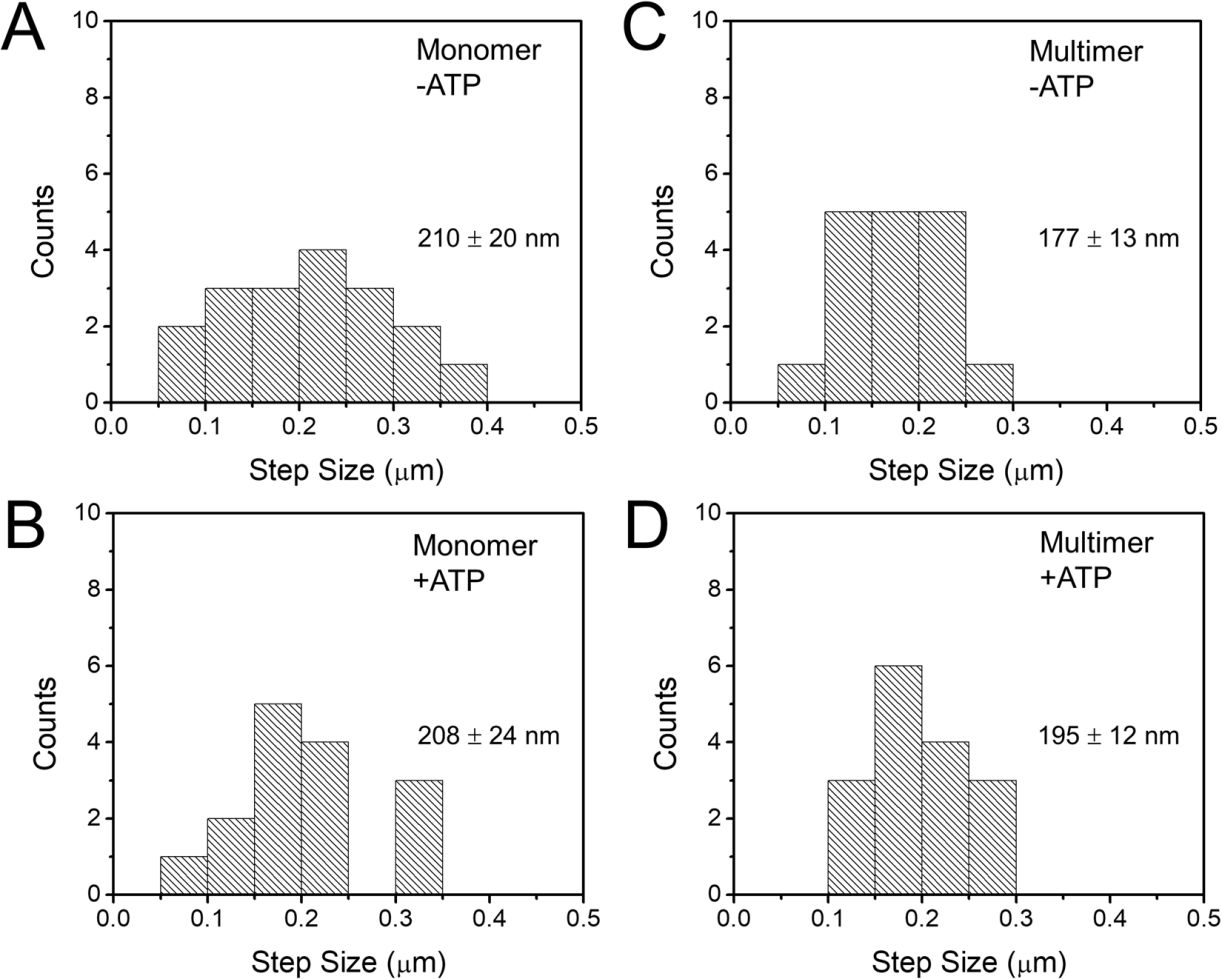
Step size distributions for DNA compaction by condensin. Discrete step sizes observed during compaction reactions were quantified, for the cases of (**A**) monomer without ATP, (**B**) monomer with ATP, (**C**) multimer without ATP, and (**D**) multimer with ATP. In each case, the step distributions were rather similar, with a range in size from roughly 50 to 300 nm and a mean step size near 200 nm (means and standard errors were: monomer –ATP: 210 ± 20 nm; monomer + ATP: 177 ± 13 nm; multimer –ATP: 208 ± 24 nm; multimer + ATP: 195 ± 12 nm). Overall there was little variation of the step size distribution with condensin monomer/multimer or –ATP/ + ATP, consistent with DNA-loop-capture by condensin being a predominantly ATP-independent process.

Individual traces such as in Fig. 3 were analyzed to glean insight into the mechanism of DNA compaction by condensin. In some cases, discrete step-like events such as in Fig. 3A were observed, while in other traces, more gradual compaction dominated. In other traces, a mix of step and non-step events was observed. We measured the step-sizes from step events in all traces as well as the fraction of step vs. non-step compaction, by identification of step events by hand, and then using step-measuring software to determine the step sizes (Fig. S5A, B). Histograms of step-sizes (Fig. 6) for both monomeric and multimeric condensin in the presence and absence of ATP were similar. In all cases, the average step sizes were between 177 and 210 nm, suggesting minimal variation between cases. Thus both monomeric and multimeric condensin showed rather similar step-size distributions under +/− ATP conditions.

We noticed that the compaction reactions proceeded by a combination of large step-like events, separated by continuous “non-step” compaction dynamics. When comparing the fraction of DNA which was compacted in step versus non-step events (Fig. S5A,B), it was also evident that the two condensin species did not differ significantly in their behavior. Condensin exhibited an average of 40 to 55% step-like events in all traces (Fig. S5C), with a large variation suggesting the differences were not statistically significant.

## Discussion

### “Monomer”-heteropentamer versus “multimeric” forms of condensin

We have presented biochemical and single-molecule biophysical analyses of the condensin complex from budding yeast. We find that budding yeast condensin is found in both the expected “monomer” heteropentamer size, and also in a fraction of significantly larger size in SEC, consistent with a “multimeric” form of the enzyme (Fig. 2A). While the precise stoichiometry of the “multimer” remains to be determined, our chromatography experiments suggest a size of two or three “monomer” equivalents.

The multimer is less stable at lower concentration, consistent with its ability to dissociate into monomeric form (Fig. 2B). The monomer and multimer species only slowly interconvert, in an ATP-independent manner (Fig. 2B–D, S1). This interconversion is so slow that it appears unlikely that multimerization is a result of the purification process. Given the multimer’s stability in our experiments as well as the general similarity of condensin in yeast to condensin in other species, we suspect that that multimeric form may be a general feature of condensin *in vivo*.

### Multimeric condensin shows ATP-stimulated DNA compaction against subpiconewton forces

Our single-molecule experiments indicate that all combinations of monomer/multimer and + ATP/-ATP reactions lead to compaction of DNAs against tensions of up to ≈ 1 pN applied forces (Fig. 3). However, for the combination of multimeric condensin with ATP the compaction is more complete, and compacts DNA by about 75%, significantly more than the ≈40% compaction obtained for monomer or –ATP reactions (Fig. 4A). While one might imagine that the higher compaction by the multimer is simply due to its larger size, this enhancement of compaction by multimer requires hydrolysable ATP (Fig. 4C). Furthermore, addition of ATP following an initial -ATP reaction does not lead to as strong compaction as when ATP is initially included (Fig. 4D). Preliminary experiments suggest that the differential compaction activity described herein is not explained by large differences in ATPase activity of monomeric and multimeric condensins. However, we cannot exclude the possibility that more modest changes in the ATP hydrolysis rate of multimeric condensin might contribute at least in part to its enhanced DNA compaction activity.

These findings contrast with strongly ATP-gated compaction reactions observed in a prior single-DNA-based study of condensin I from mitotic *Xenopus* egg extracts^16^; in that study ATP was observed to be essentially required to obtain any DNA compaction, and strong initiation of compaction was observed when ATP was added following an initial incubation of DNA with condensin without ATP. Interestingly, our results are in line with results of single-DNA experiments on budding yeast SMC1/SMC3 cohesin heterodimer as well as with *bacterial* condensins, where appreciable DNA compaction was observed in the absence of ATP^17,18,24^. The result that the multimer species of budding yeast condensin displays ATP-stimulated DNA compaction while the monomer does not lead us to conclude that the multimer is a more likely candidate for the active form of condensin *in vivo*.

### Force dependence of compaction reactions

The ≈200 nm step events that we observe during compaction (Figs 3A–D and 6) suggest that ≈600 bp loops of DNA can be “captured” by condensin, in accord with prior studies of *Xenopus* condensin I^16^, yeast cohesin heterodimers^24^ and bacterial SMC complexes^17,18^. As one might expect, DNA compaction by condensin is force-dependent, with more compaction occurring at lower force (Fig. 5). At very low forces of 0.2 pN, the reaction shows little ATP dependence, likely because rather complete compaction can occur via random loop capture at such low forces (see, for example^27^). Larger forces of 2 pN halt compaction, again as expected for a thermal-fluctuation loop-capture reaction. Interestingly, for intermediate forces of 0.45 pN, the compaction reaction by the multimer is most strongly ATP-enhanced (Fig. 5B); this level of tension is typical of that expected in stretches of DNA *in vivo* ^23^.

Our results indicate that optimal ATP-enhanced compaction occurs for the multimer against DNA tensions ≈0.5 pN, and that the compaction reaction is “stalled” by 1 to 2 pN forces. Looked at another way, our results indicate that yeast condensin can generate compaction forces up to ≈1 pN, which is sufficient tension to straighten DNA and chromatin random coils^17,26^ and to bias strand passages by topo II, while not being so large as to displace nucleosomes and other DNA-binding proteins (which typically require forces in excess of a few pN^28,29^). Thus, budding yeast condensin is driving compaction at force levels capable of moving DNA and chromatin segments around without damaging chromatin structure. We note that DNA tension generation is likely to be a key function of condensin simply because to compact chromosomes one needs to move chromatin segments, against at least the ≈0.2 pN forces associated with random thermal fluctuations, and likely against larger forces arising from molecular friction and steric obstructions to motion of chromatin segments.

### DNA torque plays little or no role in DNA compaction by yeast condensin

Surprisingly, appreciable DNA torque (the ≈10 pN.nm that occurs just before buckling and the onset of plectonemic supercoiling at ≈0.5 pN forces^23,30,31^) does not appreciably affect DNA compaction by yeast condensin (Fig. 4B). This result is in accord with similar torque-independence of DNA compaction by *Xenopus* condensin I^16^, but is strikingly different from the nearly on-off dependence of DNA compaction by yeast cohesin in experiments paralleling those reported here^24^. This lack of DNA-torque dependence in single-DNA experiments on condensin^16^ is in contrast to observations that *Xenopus* condensin has been reported to stabilize positive-writhe crossings in circular plasmids^32–34^, although the DNA was entirely relaxed at the beginning of those biochemical experiments, as opposed to being under tension in the magnetic tweezers study. Similar experiments on yeast condensin phosphorylated by Plk1/Cdc5 observed a capacity to generate positive supercoiling^13^, which we have not seen evidence for in the MT experiments of the present paper (for example, by a dependence of compaction on DNA torque). It may be that condensin is able to chirally affect DNA conformation at very low tension, but that this function is suppressed by even moderate (0.5 pN) DNA tension. Given that condensin likely has to work against forces in the pN range *in vivo*, our results suggest that chiral folding of DNA (e.g., generation of supercoiled or knotted structures with definite chirality) is not a key function of budding yeast condensin.

### Open questions: condensin function ***in vitro*** versus ***in vivo***

This study establishes that condensin can be found in different oligomerization states (Fig. 2), and that different oligomeric states have different DNA-organizing capabilities (Figs 3–4). We have not yet precisely quantified the degree of oligomerization present in our “multimer” fraction, but it is likely in the range of 2–3 “monomers” (condensin heteropentamers). Exactly what the conformation is of the “multimer” state remains to be determined, but a number of authors have suggested that SMC complexes might be active as complexes larger than a single heteropentamer^35,36^.

Our results contrast with the only other prior experiment of this type on eukaryote condensin, which used condensin I from mitotic *Xenopus* egg extracts^16^. Instead of an abrupt on-off dependence of DNA compaction on +/- ATP, we see appreciable compaction by the yeast complex even in the absence of ATP, in a manner similar to prior experiments with the yeast SMC1/SMC3 cohesin heterodimer complex^24^ as well as for bacterial SMC complexes^18,35^. It could be that there are appreciable differences in DNA-structuring functions of condensins in yeast and vertebrates, or it could be that the *Xenopus* experiments involved condensins with different conformations or post-translational modifications (perhaps associated with the pre-fertilization state of the *Xenopus* eggs versus unsynchronized yeast cells). Experiments of the type presented in this paper using varied post-translational modifications would be desirable, although achieving precisely the modifications associated with particular cell cycle states is technically challenging^13,19^.

We have ensured that the proteins from the condensin heteropentamer are the only proteins in our experiment (Fig. 2). It is quite possible condensin function might be strongly modulated by other proteins, including histones/nucleosomes on DNA, or factors associated with loading and/or unloading of the complex from DNA/chromatin. Recent biochemical and single-molecule studies of cohesin indicate that loading factors are needed to achieve encirclement of DNA by the cohesin complex^37–39^, and perhaps such chaperones are needed to obtain a conformation of condensin on DNA that will permit mitotic-like DNA compaction. Furthermore, models of compaction of DNA by condensin suggest that *unloading* may be key to looping-driven compaction^40–42^. While recent biochemical studies suggest that a minimal system for mitotic chromosome compaction might consist only of condensin, chromatin and topo II, other proteins may well play key roles in facilitating mitotic compaction.

An attractive model that has been proposed for DNA structuring by SMC complexes is that of “loop extrusion”, which supposes that SMC complexes first bind to a contiguous stretch of DNA/chromatin, and then proceed to gradually “extrude” DNA loops from the SMC-DNA complex^40–43^. Combined with intermittent dissociation and reassociation, this type of dynamical model has the capacity to generate robust compaction of chromatin into regularly sized loop domains, as has long been suggested to occur on metaphase chromosomes by electron-microscopy studies^44,45^. In the last few years results from *in vivo* Hi-C DNA-sequence-juxtaposition-analysis experiments have suggested that human cohesin^46–48^ and bacterial condensin^36,49^ may have a “loop extruding” function *in vivo*. Very recent observations of ATP-dependent directed motion of condensin along DNA also are in accord with the loop extrusion model, which assumes DNA translocase activity by condensin^50^.

**Figure 7:**
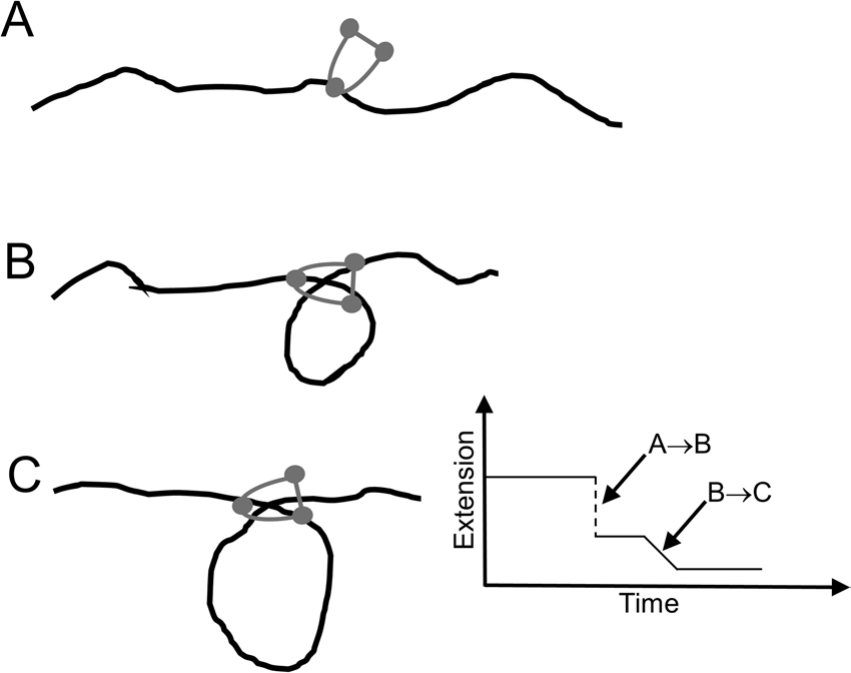
Loop-capture and loop-enlargement functions of condensin. In the single-DNA experiments condensin can mediate “loop capture” events which generate sharp jumps down in extension (A→B) of size roughly 200 nm (600 bp). Following loop capture, length can be absorbed into the loop gradually (B→C), possibly in an ATP-dependent manner, and possibly preferentially for condensin multimers (only monomers are sketched here). The step-like and smooth extrusion dynamics are highly idealized in this sketch.

A hallmark of a loop-extrusion mechanism in a single-molecule context would be gradual compaction by both step-like and slow “creeping” dynamics, with both forward *and reverse* (unfolding) dynamics. In fact, both our experiments with the multimer (Fig. 4 and Fig. S5), and those of Strick and Hirano using *Xenopus* egg extracts^16^ display these features. We suspect that the step events correspond to capture of small ≈600 bp (200 nm) loops of DNA by condensin complexes (Fig. 7A,B), and that a separate loop-size-variation dynamics generates the non-step compaction (Fig. 7B,C). It is possible that the initial step-generating loop-capture step is not ATP-dependent, given the similarity of step sizes with and without ATP present (Fig. 6). We speculate that the non-step component of the compaction dynamics may be ATP-dependent and is prevalent for the multimeric condensin form. Clearer determination of condensin functions on DNA will require direct observation of DNA conformation, using either fluorescently labeled DNA or condensin, in a combined fluorescence/magnetic tweezer experiment^51^.

## Electronic supplementary material

Supplementary Information

Supplementary Worksheet
