# Supplementary material for: Oligomerization and ATP stimulate condensin-mediated DNA compaction
Source: Sci Rep. 2017 Oct 27;7:14279. doi: 10.1038/s41598-017-14701-5 (PMC5660149; doi:10.1038/s41598-017-14701-5)
Supplement: Supplementary file 1 — Supplementary Information [file 41598_2017_14701_MOESM1_ESM.pdf]

Supplementary Information for

Oligomerization and ATP stimulate condensin-mediated DNA compaction

Ross A. Keenholtz[1], Dhanaraman Thillaivillalan[2], Roger Palou[2], Jia Yu[1,3],
Damien D'Amours[2,4] and John F. Marko[1, 5]

[1]Department of Molecular Biosciences, Northwestern University, Evanston, IL 60208, USA
[2]Institute for Research in Immunology and Cancer, Université de Montréal, Montréal, Québec H3C 3J7, Canada
[3]College of Life Sciences, Qingdao University, Qingdao, 266071, China
[4]Department of Cellular and Molecular Medicine, University of Ottawa, Ottawa, Ontario K1H 8M5, Canada
[5]Department of Physics and Astronomy, Northwestern University, Evanston, IL 60208
Corresponding author: John F. Marko
john-marko@northwestern.edu

Keywords: DNA topology, DNA compaction, single-molecule, condensin, chromatin, SMC proteins

Supplementary Figures

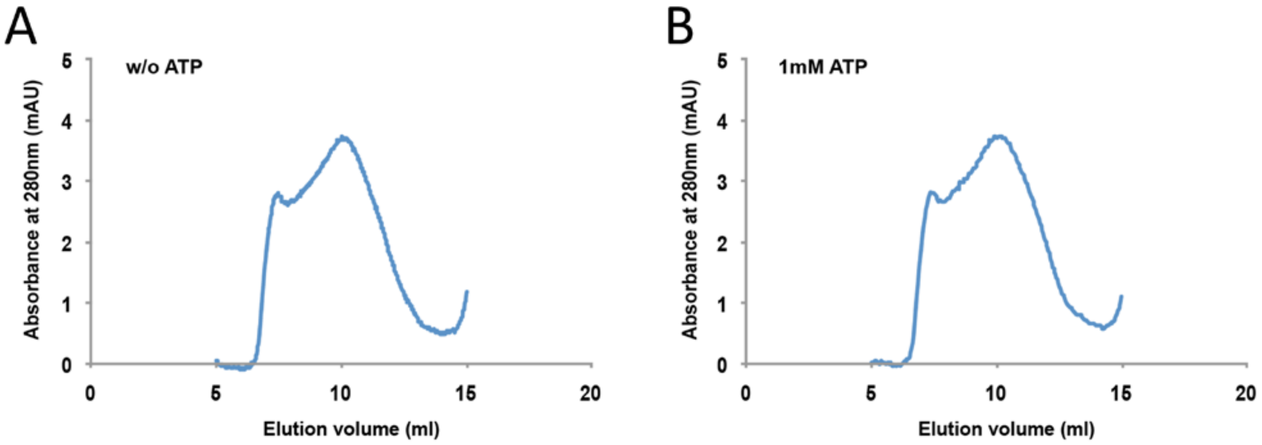

Figure S1. Effect of ATP on the oligomerization state of yeast condensin.
After purification, monomeric condensin fractions were pooled, concentrated, and divided into two parts. One part was supplemented with 1 mM ATP, whereas the other part was left untreated. The samples were incubated overnight at 4 °C to allow time for changes in condensin oligomerization state. After this incubation, samples without **(A)** and with **(B)** ATP supplementation were resolved by SEC, as previously described.

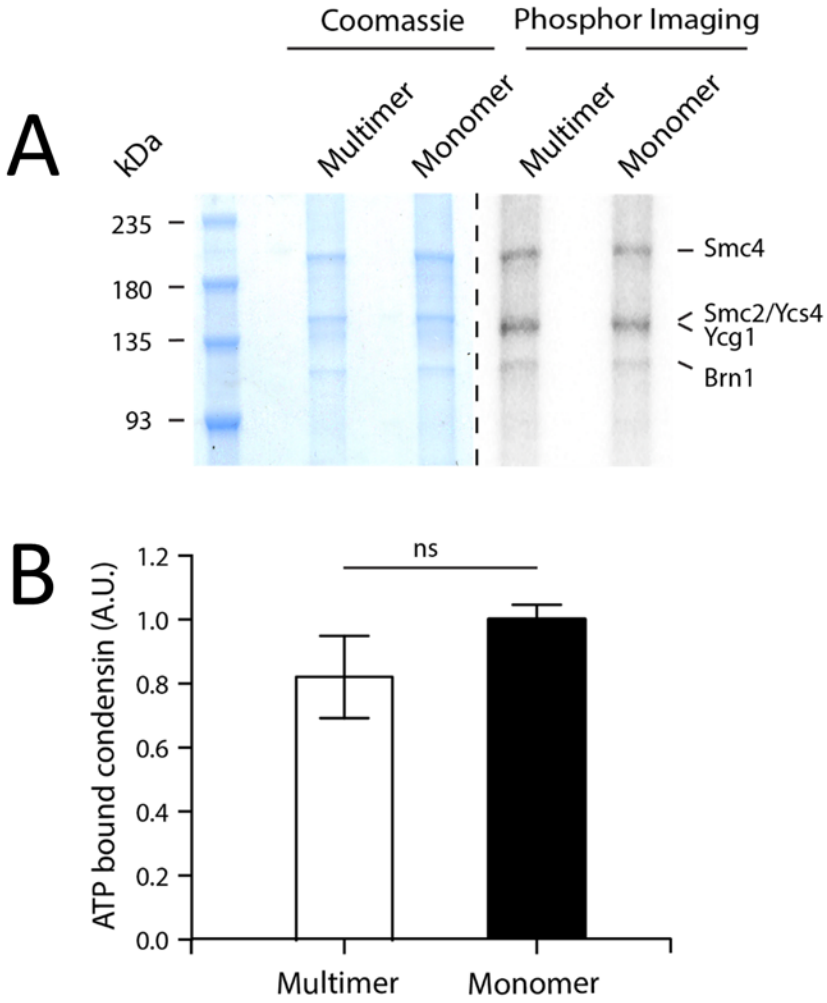

Figure S2. Effect of oligomerization state on ATP binding by condensin.
Condensin binding to ATP was determined by UV crosslinking of radiolabeled ATP to monomeric and multimeric condensin preparations.
(A) Left: Coomassie staining of multimeric and monomeric yeast condensins after UV crosslinking and electrophoresis. Right: Autoradiogram of the gel showing [^{32}P]ATP bound to condensin subunits.
(B) Quantification of ATP bound to multimeric and monomeric condensin subunits normalized for protein abundance in the SDS-polyacrylamide gel. Data are shown as arbitrary units (A.U.) and represent the average of three independent experiments (error bars: standard deviation). The difference between ATP binding by multimer and monomer was not statistically significant (ns).

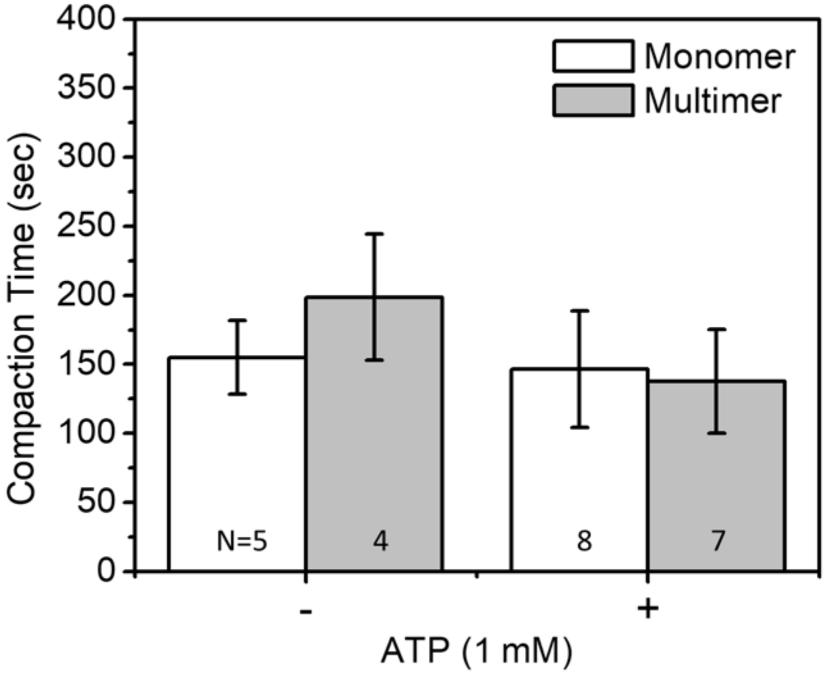

Figure S3. Kinetics of compaction reactions.

Reactions with condensin monomer and multimer, with and without ATP, had their compaction completion time determined by measuring the time required for the compaction to proceed from 10% to 90% of the final value achieved. The resulting times were averaged to obtain the bars and standard errors shown. The four classes of experiments displayed statistically the same compaction times.

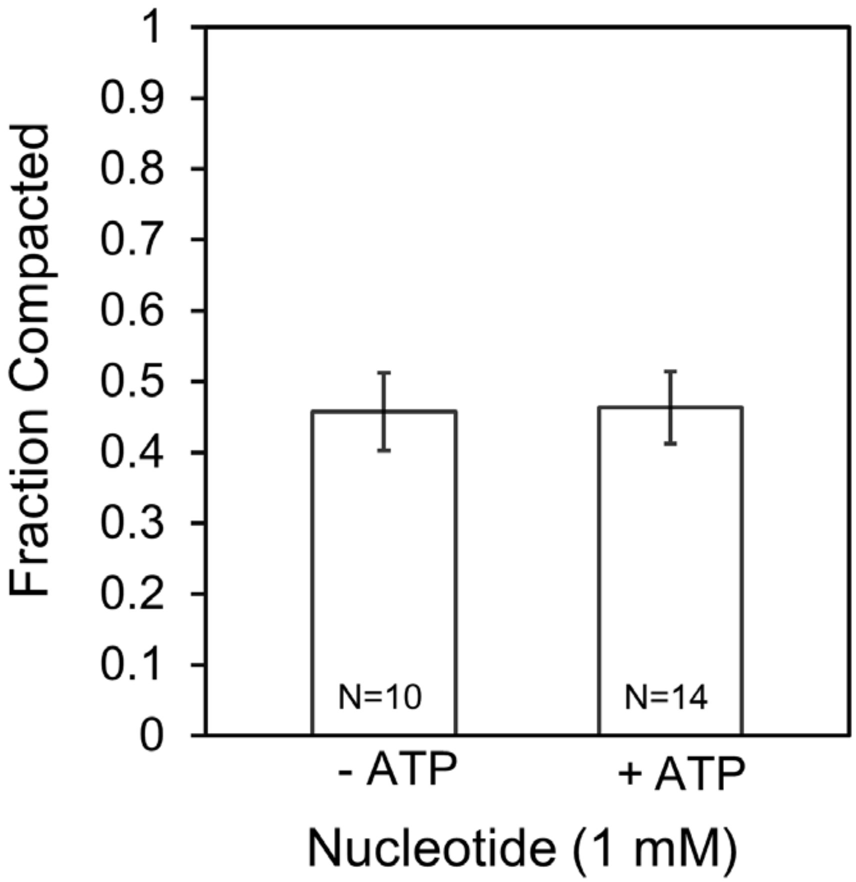

Figure S4. Compaction reactions using ATPase mutant condensin multimer.
Compaction reactions were carried out with the multimer fraction a condensin complex containing a version of Smc4 with a mutation inactivating a key residue of its Walker A motif (Lys191). Experiments were done as for wild-type condensin, with and without 1 mM ATP at an applied force of 0.45 pN. Nearly the same degree of compaction, and a compaction level similar to that for the wild-type condensin multimer without ATP were observed (see Fig. 4A), supporting the conclusion that ATP hydrolysis is responsible for the enhanced compaction observed for the wild-type condensin multimer +ATP reactions.

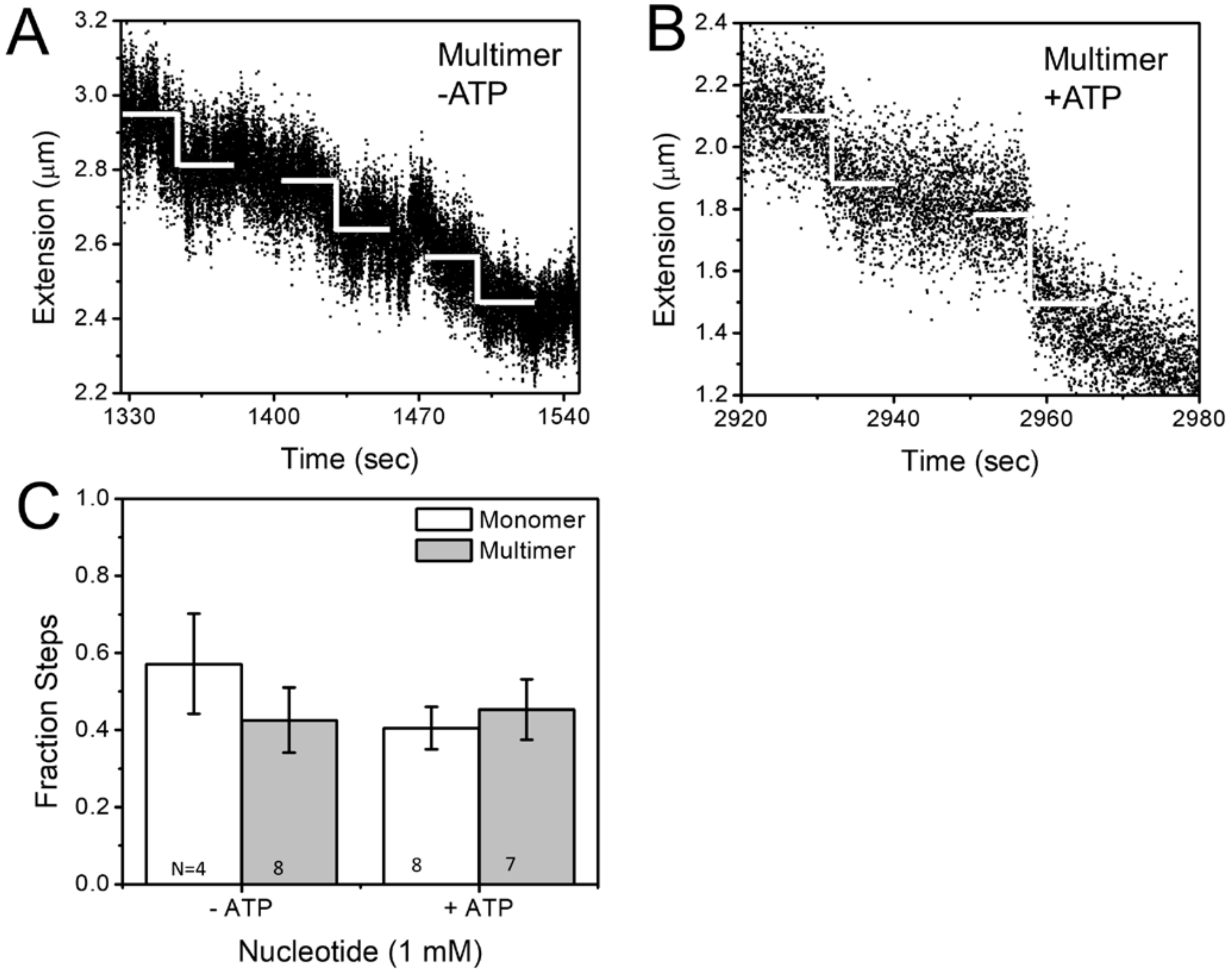

Figure S5. Fraction of step-like events.
(A-B) Representative kinetic traces are shown for **(A)** multimer –ATP and **(B)** multimer +ATP compaction reactions, showing identified steps, with non-step length change between the steps.

(C) The total fraction of compaction step-like events in single-DNA experiments was plotted for monomeric and multimeric condensin in the presence or absence of ATP. The total percentage of step-like and non-step-like events (i.e. gradual compaction) sums to 100%. The four cases show little variation in step versus non-step event composition.

Supplementary Worksheet. Data sets for DNA compaction experiments.

The pages of this worksheet give the individual measurements associated with the bar graphs in Figs. 4 and 5 of the paper. Each worksheet also presents the data for each panel as a scatterplot, so that the reader can see the distribution of individual measurements (black circles). The gray X's indicate the averages in each category (corresponding to the height of the bars in the corresponding bar graphs) while the horizontal gray bars indicate the standard error in the mean, also shown as error bars in Figs. 4 and 5.
